# Melanoma and brown seaweed: an integrative hypothesis

**DOI:** 10.1007/s10811-016-0979-0

**Published:** 2016-10-11

**Authors:** Jane Teas, Mohammad R. Irhimeh

**Affiliations:** 1 Thomas Cooper Library Fellow, University of South Carolina, Columbia, SC 29208 USA; 2 Cell and Tissue Therapies Western Australia, Royal Perth Hospital, Perth, WA 6000 Australia; 3 Faculty of Medicine, Dentistry and Health Sciences, The University of Western Australia, Crawley, Perth, WA 6009 Australia

**Keywords:** Brown seaweed, Melanoma, Epidemiology, prebiotics, Fucoidan, Fucoxanthin, Dietary brown seaweed

## Abstract

Although relatively rare, melanoma accounts for 2 % of cancer diagnoses globally and accounts for about 1 % of all cancer deaths. Worldwide, the annual incidence of melanoma is 272,000 cases which vary hugely, ranging from Japan where it is incredibly infrequent, to Queensland, Australia, where it is nearly 100 times higher. Based on epidemiology and laboratory studies, there is compelling evidence suggesting that seaweed might be protective against different types of cancers such as breast cancer in seaweed consuming populations. By comparing countries where melanoma is more common with countries where it is infrequent, it is possible to construct a hypothesis for how consuming brown seaweeds which may hold clues to the differences in melanoma susceptibility between Japanese and Western nations. Unlike in these other countries, where melanoma incidence has increased dramatically over the last two decades, in Japan, rates have remained remarkably low and stable. There is limited evidence from clinical studies and animal models that have used whole seaweed or isolated fractions from seaweed and measured changes in biomarkers. They have demonstrated the effectiveness of seaweed at inhibiting melanoma development and progression. In this review, the various results will be described. Although there are several effective fractions, it is proposed that consuming whole seaweeds may hold additional benefits that could be lost by consuming only a single extract.

## Introduction

Melanoma is a relatively rare skin cancer in most of the countries of the world, accounting for about 2 % of all cancers, and is responsible for 1 % of cancer deaths (Global Burden of Disease Cancer 2015). It has been called the most aggressive of all human cancers (Chin et al. 1998). Globally, an estimated 272,000 people are diagnosed with melanoma (Global Burden of Disease Cancer 2015; World Health Organization 2016). However, the incidence rates vary profoundly. In Japan, less than one person per 100,000 is diagnosed with melanoma, compared with Australia where current estimates for women are 30/100,000 and for men are 40/100,000 (Queensland Government 2015). Incidence rates have increased in many countries, but not in Japan. Five-year survival rates for people with melanoma depend on the stage of the disease at the time of diagnosis. If treated early, there is a high rate of cure, but for patients with late stage disease the success rate for therapies, even the new immunotherapies, is about 15–20 % at 5 years (American Cancer Society 2016). The development of treatment resistance suggests that much remains to be understood about how melanoma can remain dormant for years, avoiding detection by the body’s immune system.

Melanoma has been reported in dogs, horses, pigs, birds, and fish (Sweet et al. 2012; Williams et al. 2012). It also seems to be part of our human heritage, although found most commonly among fair-skinned people with red hair. The oldest record of melanoma dates back 2400 years ago to a mummy in Peru (Rebecca et al. 2012). About the same time in Greece, Hippocrates described melanoma when he wrote about “fatal black tumors with metastasis” (Chin et al. 1998). Generally, the modern risk factors include fair skin, light-colored eyes, red hair, inability to tan, intermittent exposure to strong sunlight, especially blistering sunburns during childhood, and family history of melanoma (World Health Organization 2016). In the last 100 years, the incidence of melanoma has dramatically increased. This has been largely attributed to changing attitudes towards having tanned skin. Coco Chanel, a French fashion designer and cultural icon in the 1920s, popularized tanned skin as glamorous and it became associated with having leisure time associated with wealth and high social status (Erdmann et al. 2013). Generally, this image of a healthy tan has persisted, particularly where people no longer do outside manual labor, such as farming. The use of tanning beds during cold dark winters continues despite recent scientific evidence that their use increases the risk of developing melanoma (Lazovich et al. 2010). People in Asia have a very different attitude towards tanning, and in general, avoid sun exposure. Pale skin is preferred which can be seen in the high demand for cosmetics that lighten skin (Quah et al. 2014).

Although much has been discovered about the genetic basis for melanoma, a cure for this cancer remains elusive. The development of melanoma is a complex multistage process, including a three-step initiation–promotion–progression system mediated by several cellular, biochemical, and molecular alterations (Rocha et al. 2007). Melanocytes are derived from pluripotent neural crest stem cells and are responsible for melanin production in specialized melanosomes. The pigment produced leads to variations in color of skin, hair, feathers, eyes, and scales in all vertebrates (Mort et al. 2015). Melanocytes and their development is modulated by the KIT (CD117) and microphthalmia-associated transcription factor (MITF), and is involved in pigmentation and cell death, which also plays a major role in the development and differentiation of melanoma, migration of melanoma cells, and drug resistance (Yajima et al. 2011; Lau et al. 2015). MITF is also involved in regulating the amount of melanin produced by melanocytes, thereby a prime target for treatments that aim to lighten skin.

Sunburns may be a major factor in reducing melanoma risk, however evidence has accumulated that diet may also play a role. Melanoma risk is positively associated with obesity as is a progression in diet-induced obesity (Chen et al. 2013; De Pergola and Silvestris 2013). People in Japan have a fraction of the rate of obesity (3.2 %) as that reported in high melanoma countries such as the USA (30.6 %), Australia (21.7 %), and New Zealand (20.9 %) (Organization for Cooperation and Development 2005). Diet choices have fueled this increase in obesity, with larger portion sizes, higher caloric intake, and increased intake of refined carbohydrates and fats (Tokudome et al. 2000). However, studies of high-fat diets have revealed contradictory data. A healthy low-fat American diet seems to be protective, but a change from a high-fat diet to a low-fat (<20 %) diet with increased fruit and vegetables and grain was associated with an increased risk of melanoma in a large study of nurses in the USA (Gamba et al. 2013). A study in Australia, where the incidence of melanoma is the highest in the world, likewise found suggestive evidence that a high-fat diet was not associated with high risk of melanoma and might even be protective (Granger et al. 2006).

The range of food eaten may be important. The Japanese diet is noted for its wide assortment of vegetables, fish, shellfish, and drinking tea, and thus is similar to eating a traditional Mediterranean diet, which is associated with about a 40 % decrease in the risk of melanoma (Fortes et al. 2008). This protective effect of a healthy diet was also observed in a second study, although the specific effects of a Mediterranean diet were limited to women under the age of 50 (Malagoli et al. 2015). There have been no studies of diet and melanoma in a Japanese population.

Several studies have previously evaluated the use of specific diets to treat melanoma patients. One retrospective comparative analysis reported a six-fold increase in five-year survival rates of melanoma patients treated with the Gerson diet (low sodium, high potassium, lactovegetarian diet that emphasizes fresh vegetables, fruit juices, and vitamin supplements). However, the flawed methodology limits the reliability of this study, but the results are intriguing (Hildenbrand et al. 1995).

Due to the increasing incidence and elusive cure for melanoma, there has been increasing interest in natural compounds that may hold promise (Chinembiri et al. 2014). Collins and his colleagues have reviewed the potential uses of seaweed extracts in a range of modern life diseases, but again, it does not include the consumption of whole seaweeds in a whole diet (Collins et al. 2016). Mohamed and colleagues have reviewed the health benefits of seaweed as a sustainable functional food for use in complementary and alternative therapies (Mohamed et al. 2012).

Dietary seaweed is one of the many differences between Western diets and a traditional Japanese diet. Given its unusual distribution as a culinary favorite, it is easy to speculate that including seaweed might make a difference. On average people in Japan eat 14.3 g day^−1^ of seaweeds (Fukuda et al. 2006). The actual amount of seaweed is difficult to estimate since it is consumed as a hot water extract in soup, as a flavoring for other foods (dashi), as a condiment, as a side dish, and occasionally as part of an entrée. It is also commonly eaten as a snack. Currently, dietary seaweed is increasing worldwide, and it has been called the “superfood” by several prominent chefs including the British chef Jamie Oliver in his most recent book (Oliver 2015).

Several difficulties are associated with the study of seaweeds against any human disease. First, there are about 30,000 seaweeds, although only five Konbu (*Saccharina japonica*), Wakame (*Undaria pinnatifida*), Hijiki (*Sargassum fusiforme*), Nori (*Pyropia tenera*), and Mozuku (*Nemacystus decipiens*) are commonly eaten in Japan (Fukuda et al. 2006). Additionally, of the 267 studies using seaweeds in cancer studies (indexed by PubMed, 5 August 2016), many have used either a proprietary blend or rarely eaten seaweed, perhaps in hopes of identifying a patentable extract.

### Seaweed specific studies of inhibition of melanoma

Seaweeds have been studied in vitro and in vivo and several extracts have shown efficacy at the inhibiting formation of many forms of cancer. Although there have been more than 250 studies of seaweeds and cancer, few have focused on melanoma (PubMed, 5 August, 2016). These studies are presented in Table 1. Melanoma cells are relatively easy to grow in cell culture and in animal models. They readily metastasize providing informative models for human melanoma. However, the applicability of results from cell culture to humans is tentative. Many of the natural defense mechanisms of the complex human body play an important role in mitigating the effects of carcinogens and anti-carcinogens. Likewise, the commonly used mouse melanoma model appears to be good at studying the metastatic behavior of melanoma cells but lacks the mechanisms of UV initiation and modulation of the cancer process. It is therefore extremely attractive to examine population patterns of melanoma to discern information about melanoma in human.

**Table 1:** Comparison of brown seaweed extracts with activity against melanoma

Seaweed extract	Seaweed	Activity	Reference
Ascophyllan			
	*Ascophyllum nodosum*	↓ migration and adhesion in vitro ↓ lung metastases in vivo ↑ natural killer cell activity	Abu et al. 2015
Fucoidan			
	*Sargassum* sp. *Fucus vesiculosus* (from Sigma)	↓ melanoma cells dose dependent ↑ apoptosis ↑ natural killer cell activity	Ale et al. 2011a
	*Sargassum henslowianum* *Fucus vesiculosus*	Dose response ↓ melanoma cell proliferation *Sargassum* ˃ at lower dose; ˃ *Fucus* at higher dose Both ↑ apoptosis via ↑ caspase-3	Ale et al. 2011b
	*Chordaria flagelliformis*	↑innate immune system via CD11c integrins	Anisimova et al. 2015
	*Alaria sp.* *Saccharina japonica*	↓ proliferation and colony formation; Reproductively active tissue had highest anti-melanoma activity	Vishchuk et al. 2012
	*Fucus vesiculosus*	As adjuvant tumor vaccine (with chicken ovalbumin) induced Th1 and C-type lectin response, protecting mice from melanoma	Jin et al. 2014
	*Fucus vesiculosus*	Fucoidan and oversulfated fucoidan ↓ melanoma growth and angiogenesis in mice	Koyanagi, 2003
	*Saccharina latissima*	↓ angiogenesis and tumor weight ↓ microvessel density	Croci et al. 2011
Fucoxanthin			
	*Saccharina japonica*	↓ expression and secretion of MMP-9 and glycoprotein CD44 and ↑CXCR4 (important in tumor adhesion, extracellular invasion and migration) in vitro; ↓ number tumors in vivo	Chung et al. 2013
	*Fucus evanescens*	↓ growth human melanoma cells dose dependent manner; 114 μM effective concentration	Imbs et al. 2013
	*Ishige okamurae*	↑ cell cycle arrest G(0)/G(1) phase; ↑ apoptosis and protein levels of Bcl-xL ↑ caspase-9, caspase-3, and PARP; ↓ growth of melanoma tumor masses in mice	Kim et al. 2013
	*Saccharina (Laminaria) japonica*	↓ tyrosinase; ↓ suppressed skin mRNA expression related to melanogenesis in melanoma tumors in mice	Shimoda et al. 2010
	*Undaria pinnatifida* Fucoxanthin (from Sigma)	↓ melanoma cell growth	Wang et al. 2014
Galactolipids			
	*Fucus evanescens*	↓ growth human melanoma cells dose dependent manner; 104 μM effective concentration	Imbs et al. 2013

The best known of the anti-carcingenic seaweed extracts is fucoidan. Several animal studies show fucoidan inhibits melanoma in vitro and in vivo (Koyanagi et al. 2003; Ale et al. 2011a, 2011b; Croci et al. 2011; Vishchuk et al. 2012; Jin et al. 2014; Anisimova et al. 2015). These are presented in Table 1 *. C*umashi et al. (2007) reviewed the many anti-inflammatory, anti-coagulant, anti-angiogenic, and anti-adhesive activities of fucoidans, and although varying slightly between the nine species examined, generally concluded that fucoidans could be helpful in improving health. A study supporting a role for fucoidan in photoprotection was done by Anisimova et al. (2015). They reported a 1.9-fold increase in cytotoxic activity of spleen mononuclear leucocytes towards melanoma cells of line B16 and concluded that one of the ways seaweed fucoidan could influence antitumor activity was as an effector of the innate immune system via CD11c integrins. Fucoidan has been studied as both an anti-carcinogenic agent on its own and as an adjunct to conventional therapies (Kwak 2014; Abu et al. 2015; Atashrazm et al. 2015). A study of 70 elderly subjects who were presumed to have age-related immune suppression were randomized to either a daily dietary supplement of 300 mg fucoidan or placebo given as an adjuvant to standard seasonal influenza vaccine. There was a significant enhancement of natural killer cell activity associated with fucoidan but no increase in the group given the placebo (Negishi et al. 2013). As the most effective treatments for late stage melanoma involving immunotherapy that results in increasing natural killer cell activity, this study is supportive of a role for dietary fucoidan in melanoma treatments.

Several studies have reported that seaweed extracts inhibited melanogenesis in mouse melanoma cells, and should be considered for use in cosmetics for skin lightening (Kim et al. 2013; Jin et al. 2014; Song et al. 2015). Since lighter skin is a risk factor for susceptibility to melanoma, the activity of fucoidan may not be helpful. However, Maruyama and colleagues reported that dietary *Undaria* sporophyll (mekabu) was associated with significant suppression of the inflammatory response to UVB exposure in mice (Maruyama et al. 2015). Perhaps it could be both an inhibitor of melanogenesis and its pro-inflammatory reaction to UV light as well as a photoprotector against the DNA damage that may cause melanoma.

A study of a polysaccharide compound from *Ascophyllum* sp., reported to be different in chemical composition from fucoidan, has also been reported to have immune stimulating activity against melanoma (Abu et al. 2015). Invasion and migration of melanoma tumor cells were inhibited in vitro and lung metastases were inhibited in vivo*.*

Dietary carotenoid intake has been associated with reduced risk of melanoma (Millen et al. 2004). Fucoxanthin is the major form of carotenoid found in brown seaweed chloroplasts, where it is part of the light-harvesting complex algae use for photosynthesis and photoprotection. It accounts for about 10 % of all natural carotenoids found in nature (Miyashita et al. 2011). Animals cannot synthesize carotenoids but must obtain them from their diet. Fucoxanthin has an unusual shape with an allenic bond found in only about 40 of the more than 700 different naturally occurring carotenoids. This may account for its wide range of biological activities including thermagenesis, anti-obesity, anti inflammatory, and anti carcinogenicity (Miyashita et al. 2011; Kumar et al. 2013; Mikami and Hosokawa 2013). The data for specific anti-melanoma activity include four studies demonstrating significant inhibition of melanoma in vitro and in vivo (Shimoda et al. 2010; Chung et al. 2013; Imbs et al. 2013; Kim et al. 2013; Thomas and Kim 2013; Wang et al. 2014). Although not specifically melanoma studies, several authors have reported fucoxanthin (either orally or topically applied) reduced UVB-induced skin inflammation in vitro and in vivo, and was important in skin whitening (Lee et al. 2013).

Phlorotannins are phenolic compounds that are part of the structural components of brown algae cell walls. They are produced by seaweeds in response to high UV-B radiation and grazing threats. They have been studied as possible sources of sunscreens and other beneficial bioactive compounds (Li et al. 2011). In particular, phlorotannins have been studied as tyrosinase inhibitors in melanoma cells although the intent of the studies was to identify skin lightening compounds in seaweeds, rather than to act against melanoma tumors (Yoon et al. 2009; Kang et al. 2012).

Prebiotics have recently emerged as an important defense against cancer. In humans, the intestine hosts about 10^14^ bacteria, or about ten times the number of human cells in an average human (Gerritsen et al. 2011). The role for intestinal bacteria in melanoma has recently emerged. In a study of two groups of rats given a melanoma immunotherapy (programmed cell death-1 inhibitor) (PD-1 blocker), it was discovered that rats with gastrointestinal populations of *Bifidobacterium longum* had the same tumor control as those rats administered the anti-PD-1 therapy that was being studied. Oral administration of *Bifidobacterium* to rats without this intestinal bacteria was effective in inducing the same response, and the combination of *Bifidobacterium* and anti-PD-1 therapy almost abolished tumor growth (Sivan et al. 2015).

Dietary seaweeds have been found to have significant prebiotic effects that influence *Bifidobacterium* populations (de Jesus et al. 2016). Wang reported that 2.5 % alginate supplementation in rats increased *Bifidobacterium* by 13-fold compared to the control group (Wang et al. 2006). An et al. (2013) investigated the effects of two of the primary fermentable seaweed polysaccharides on the relative abundance of microbiota in the caecal contents of rats fed a control (no fiber), a 2 % alginate diet, and a 2 % laminarin diet. They reported that alginate was associated with a four-fold increase from 1 % in the relative abundance of the bacteria in the *Actinobacteria* phylum but a decrease to 0 % in the laminarin supplemented the diet. *Bifidobacterium*, although a member of the Actinobacteria phylum, was not specifically identified. Interestingly, red seaweeds appear to have a similar effect in increasing *Bifidobacterium* in animal studies using 2.5 % *Chondrus crispus* (4.9-fold increase) in a rat study (Liu et al. 2015). A 1 % supplementation with *Sarcodiotheca gaudichaudii* in a chicken study was associated with a 14-fold increase (Kulshreshtha et al. 2014).

Alginic acid has been used for decades as an over the counter treatment for stomach hyperacidity (Malmud et al. 1979; Holdt and Kraan 2011). The mode of action is to combine with stomach acid to form a floating raft above the stomach contents, thereby reducing stomach acid reflux. A study of healthy male volunteers reported that a daily intake of 10 g day^−1^ of alginate was associated with a significant increase in fecal *Bifidobacteria* (Terada et al. 1995). In a general comparison of fecal excretion of diverse bacteria, 106 healthy Japanese volunteers had almost 20-fold higher excretion of *Bifidobacteria* than people from 11 other countries studied (Nishijima et al. 2016). These studies provide circumstantial evidence for a possible role of dietary seaweed in enhancing *Bifidobacteria* populations in people who consume brown seaweeds, possibly contributing to protection against melanoma.

It is also possible that eating brown seaweed increases available fucose (Cao 2015). Protein fucosylation is key to immune cell recognition, cell signaling, and general health, and reduced cell surface fucosylation sites on melanoma cells have been reported (Lau et al. 2015). Lau and his colleagues reported that oral L-fucose (drinking water) fed to mice with melanoma restored fucosylation in the melanoma tumors leading to about a 300 % increase in the number of intratumoral natural killer cells and decreased metastases. In the same study, the authors reviewed tumor tissue samples from 320 human melanoma patients. Higher expression of melanoma cell surface protein fucosylation was associated with improved overall survival and 34 % lower risk of metastases. The exact mechanisms of action are still being investigated. Bioavailability studies of brown seaweeds have focused on fucoidan, rather than fucose (Irhimeh et al. 2005; Warttinger et al. 2016). Fucose availability after eating raw seaweed is negligible but if seaweed is digested by heat or enzymes, as is likely with dietary seaweed, or gut microbes following ingestion, then free fucose would be made available. The fate of fucose in the gut is to cross the membranes by diffusion or to bind with certain cells (M) in Peyers’s patches and modulate the immune system**.**

### Clinical studies of brown seaweeds in healthy people

Accordingly, maybe another way to look at brown seaweeds and melanoma is a general picture of what whole seaweed does in healthy people and animals. We looked at healthy humans in several clinical studies and have found that the inclusion of 5 g day^−1^ brown seaweed (*Alaria esculenta* and *Undaria pinnatifia*) was associated with changes in biomarkers found in melanoma (Teas et al. 2009; Teas et al. 2011; Teas et al. 2013). Fifteen healthy postmenopausal women consumed 5 g day^−1^ placebo for a month, followed by 5 g day^−1^ *Undaria* for a month, followed by 5 g day^−1^ of placebo for a month. The most profound change was a significant 50 % reversible reduction in urinary urokinase receptor (uPAR). In most cancers, patients with metastatic disease have increasing excretion of uPAR and this is prognostic of a rapidly progressing disease. uPAR is an important factor in inflammation, differentiation, proliferation, detachment from the extracellular matrix, and migration. Although we observed this change in healthy women, they were mostly overweight (average body mass index (BMI) of 30, which is the lower limit for being obese). Adipose tissue has been reported to be associated with an ongoing turnover of uPAR (Choe et al. 2016). However in terms of relevance to melanoma, in vitro and in vivo studies of reducing uPAR on the surface of tumor cells were associated with inducing a state of dormancy (Yu et al. 1997; Ossowski and Aguirre-Ghiso 2010; Noh et al. 2013). It remains to be seen if seaweed ingestion by melanoma patients could have a similar effect.

In a different study, we found a decrease in p-selectin plasma concentration. In a small pilot study of eight people challenged with a high fat breakfast with and without 5 g *Undaria*, we found that 4 h after eating that there was a significant difference in p-selectin. It was increased with placebo and decreased with seaweed (unpublished data, poster). For various reasons, this study was not repeated in a larger sample size, but a plethora of studies in animals have reported that dietary seaweed and its extracts decrease p-selectin (Fitton 2011; Fitton et al. 2015). Many cancer cells including melanoma tumor cells bind to p-selectin in the process of moving from one site to another. This effectively hides the tumor cells from the body’s immune attack and from the sheer stress of blood flow around them, increasing the likelihood of extravasation into new tissue sites for metastases.

### Limitations of studying of seaweeds

Several difficulties are associated with the study of seaweeds against any human disease. First, there are about 30,000 seaweeds, although only five Konbu (*Saccharina japonica*), Wakame (*Undaria pinnatifida*), Hijiki (*Sargassum fusiforme*), Nori (*Pyropia tenera*), and Mozuku (*Nemacystus decipiens*) are commonly eaten in Japan (Fukuda et al. 2006).

The structure and activity of various components of seaweed fractions have been widely studied. However, there are few standard protocols for methods for extraction leading to some lab-specific differences (Ale et al. 2011c; Jin et al. 2016; Olivares-Molina and Fernández 2016). Other sources of variability in studies of seaweed include pre-harvest variables such as differences reported for the same kind of seaweed harvested in different geographic environments, season, reproductive status of the part of the plant used, part of the plant used, age of the plant, as well as postharvest handling, such as washing with seawater or fresh water, boiling, salting, shade or sun drying, as well as storage (Vishchuk et al. 2012; Mak et al. 2013; Ehrig and Alban 2015). Each of these factors has been shown to impact biological activity.

## Conclusions

In Japan, where most people consume seaweed daily, exceptionally low incidence and mortality rates of melanoma are reported. These rates have remained stable over decades, whereas in other developed countries rates have steadily increased. Seaweeds contain many interesting compounds, some of which have been studied specifically in conjunction with melanoma and found to actively inhibit tumor initiation, proliferation, and progression. However, due to the many sources of variability in seaweed quality, it may be important to eat a variety of whole seaweeds rather than to isolate individual extracts. People in Japan eat a variety of whole seaweeds, not extracts. It seems likely this approach may be better than attempting to find a specific extract or fraction to commercially exploit as a “magic bullet” at the expense of possibly omitting an important component. Using a mixture of whole seaweeds, either as food or in a supplement rather than an extract would also allow melanoma patients to add this food to their diet without regulatory approval. The need for dietary options for patients who have been told to “go home and not worry” is appealing. Dietary seaweed is already encouraged by the Ministry of Health in Japan as a healthy everyday food. Further studies in melanoma patients are warranted.
